# Rigid intramedullary nail fixation of femoral fractures in adolescents: what evidence is available?

**DOI:** 10.1007/s10195-013-0270-y

**Published:** 2013-09-29

**Authors:** D. S. Angadi, D. E. T. Shepherd, R. Vadivelu, T. Barrett

**Affiliations:** 1 West Suffolk Hospital, Bury St Edmunds, UK; 2 University of Birmingham, Edgbaston, Birmingham, UK; 3 Birmingham Children’s Hospital, Steelhouse Lane, Birmingham, UK

**Keywords:** Adolescent, Femoral, Fracture, Rigid, Intramedullary, Nail

## Abstract

**Background:**

Femoral fracture in adolescents is a significant injury. It is generally agreed that operative fixation is the treatment of choice, and rigid intramedullary nail fixation is a treatment option. However, numerous types of rigid nails to fix adolescent femoral fractures have been described. Hence, the aim of this paper was to collate and evaluate the available evidence for managing diaphyseal femoral fractures in adolescents using rigid intramedullary nails.

**Materials and methods:**

A literature search was undertaken using the healthcare database website (http://www.library.nhs.uk/hdas). Medline, CINAHL, Embase, and the Cochrane Library databases were searched to identify prospective and retrospective studies of rigid intramedullary nail fixation in the adolescent population.

**Results:**

The literature search returned 1,849 articles, among which 51 relevant articles were identified. Of these 51 articles, 23 duplicates were excluded, so a total of 28 articles were reviewed. First-generation nails had a high incidence of limb length discrepancy (Küntscher 5.8 %, Grosse–Kempf 9 %), whilst second-generation nails had a lower incidence (Russell–Taylor 1.7 %, AO 2.6 %). Avascular necrosis was noted with solid Ti nails (2.6 %), AO femoral nails (1.3 %) and Russell–Taylor nails (0.85 %). These complications have not been reported with the current generation of nails.

**Conclusions:**

Rigid intramedullary nail fixation of femoral fractures in adolescents is a useful procedure with good clinical results. A multiplanar design and lateral trochanteric entry are key to a successful outcome of titanium alloy nail fixation.

## Introduction

Femoral fractures account for 1.4 % [1] to 1.7 % [2] of all fractures in children. The incidence of femoral fractures in children has been reported as 20–33 per 100,000 per year [1, 3–5]. The common modes of injury resulting in femoral fractures in adolescents include road traffic collisions, sports injuries and falls from height [3, 6, 7]. The risk of sustaining a femoral fracture is higher in boys than in girls [3, 5, 6, 8]. Management of femoral fractures in the adolescent population poses unique challenges due to the relative sizes of the femur and the open physes [7, 9]. Treatment options of these complex injuries have evolved over the last few decades [7, 10, 11]. Operative fixation techniques described in the current literature include external fixation, open reduction and internal fixation with plate, minimally invasive plate osteosynthesis (MIPO), and flexible and rigid intramedullary nails [7, 11–13].

Rigid intramedullary nailing has been suggested as a treatment option in these patients due to the perceived advantages of stable fixation with higher union and low complication rates [9, 14]. However, several studies have questioned this approach given the risks of avascular necrosis [15–18] and proximal femoral valgus deformity [19, 20]. Some authors have explored the lateral aspect of the greater trochanter to minimise these serious complications and obtain a good outcome [21, 22]. Hence, the aim of this paper was to collate and evaluate the available evidence for managing diaphyseal femoral fractures in adolescents using rigid intramedullary nails.

Numerous types of rigid nails have been described to treat adolescent femoral fractures. Due to the lack of consistency in reported outcomes, a comparison of various rigid nails is difficult and subject to observer variability. Based on their initial experience, Flynn and colleagues proposed criteria to grade the clinical outcome following fixation of femoral fractures [23]. They classified a major complication and/or lasting morbidity, a limb length discrepancy of >2.0 cm and malunion of >10° as a poor result. However, there is no agreement in the literature regarding similar outcome criteria for rigid intramedullary nail fixation [7, 9, 11]. Initial papers [14, 24, 25] on this topic reported on the radiographic parameters described by Edgren [26]. On the other hand, some studies have not reported on the radiological outcomes [16, 27, 28]. Therefore, to ensure that we performed an objective comparison of different nails, we analysed the reported clinical outcomes from the various studies with respect to major complications (avascular necrosis, limb length discrepancy >2.0 cm, malunion >10°).

## Materials and methods

### Study identification

A current literature search of all available evidence was undertaken using the healthcare database website (http://www.library.nhs.uk/hdas). The databases searched were Medline, CINAHL, Embase, and the Cochrane Library.

A Medline search was performed using boolean statements and the wildcard symbol (*) with the following search criteria: “(femur* OR femoral*) AND (shaft* OR diaph*) AND fracture* AND (child* OR pediat* OR paediat* OR adolescent*) AND (intramedullary* OR rod* OR nail*)”. An Embase search was performed using boolean statements and the wildcard symbol ($) with the following search criteria: “(femur$ OR femoral$) AND (shaft$ OR diaph$) AND fracture$ AND (child$ OR pediat$ OR paediat$ OR adolescent$) AND (intramedullary$ OR rod$ OR nail$)”. The CINAHL database was searched using the following criteria: “(femur* OR femoral*) AND (shaft* OR diaph*) AND fracture* AND (child* OR pediat* OR paediat* OR adolescent*) AND (intramedullary* OR rod* OR nail*)”. A review of the Cochrane database for relevant articles was performed using the search criteria ‘adolescent’ AND ‘femur’ AND ‘intramedullary nail’ OR ‘intramedullary rod’.

### Eligibility criteria for the studies

Prospective and retrospective studies from the international literature describing rigid intramedullary nail fixation in the adolescent population were identified. Articles that reported on the description of the nail and/or design, clinical results and complications (specifically: avascular necrosis, limb length discrepancy and malunion) were included. Additionally, the bibliographies of all selected articles were scrutinised for relevant articles. Isolated case reports of complications following rigid nail fixation were excluded. Articles that only examined the flexible nail technique and results of its use, based on their title and abstract, were also excluded.

### Classification of rigid intramedullary nails

In this review, we adopted the classification of rigid intramedullary nails based on the generation of the design and material technology [29]:

- First generation: intramedullary nail with a cloverleaf design that resulted from pioneering work by Gerhard Küntscher [30], and was manufactured from stainless steel.
- Second generation: the length and rotation of the intramedullary nail could be controlled through the use of a bicortical screw, which led to an expansion in the indications for this technique; examples include Küntscher interlocking, Grosse–Kempf, and Russell–Taylor nails.
- Third generation: research into nail failures led to an improved design with interlocking screws manufactured from fatigue-resistant titanium alloys that were placed in a multiaxial direction.
- Current generation: multiplanar nails incorporating improvements in design and material technology from the previous three generations.

## Results

The literature search returned 1,849 articles, among which 51 relevant articles were identified. Of these 51 articles, 23 were duplicates, so 28 articles were reviewed (Table 1). A search through the bibliographies of the above articles identified a further 3 relevant articles which were also included in the analysis [27, 31–33]. A summary of the various studies on rigid nails identified from the literature search is presented in Table 2. We identified 2 papers from Ramseier [34, 35] and 1 paper from Stans [36] which discussed the results of rigid nail fixation, but these papers lacked descriptive detail regarding the type of rigid nail used. Hence, we included only the larger of the two studies from Ramseier [34] for analysis.

**Table 1:** Results of the literature search

	Database				Total
	Medline	Embase	CINAHL	Cochrane	
Search results	794	787	240	28	1,849
Relevant articles	23	24	3	0	51
Duplicates					−23
					28

**Table 2:** List of relevant studies in the literature

Author	Type of nail	Patients	Age range (years)	Average weight (kg)	Average follow-up (months)	Major complications reported? (Y/N)	Level of evidence
Reynolds et al.	Adolescent lateral femoral nail	15	10–16	59.6	9.1	N	2b
Park et al.	AO humeral nail	23	8.2–16.1	49.4	21	N	2b
Park et al.	Unreamed tibial and Sirus femoral	21	11–17.2	51.2	22.4	N	1b
Garner et al.	N/A	15	14.3–16.4	60.4	13.6	Y	2b
Ramseier et al.	N/A	37	11–17.6	55.2	14.6	Y	2b
Keeler et al.	S&N humeral	78	8.2–18.4	70	24.8	N	2b
Jencikova-Celerin et al.	Biomet interlocking	58	9.9–14.1	47.4	13.1	N	2b
Mehlman et al.	Synthes locked tibial	6	9–15	69.1	17	N	4
Kanellopoulos et al.	R-T & Targon	20	11–16	N/A	29	N	2b
Gordon et al.	S&N humeral	15	8.2–17.1	N/A	35.3	N	2b
Letts et al.	AO/G-K/R-T	54	11.4–17.1	60	63	Y	2b
Tortolani et al.	R-T adult tibial	9	11–15	N/A	24	N	2b
Townsend et al.	R-T/Delta II	34	10.2–17.6	N/A	24	N	2b
Momberger et al.	Zimmer titanium/R-T/steel	48	10–16	N/A	16.2	N	2b
Stans et al.	N/A	13	11.1–16.2	50.2	19	Y	2b
Buford et al.	Solid titanium	54	6–15	N/A	20	Y	1b
Skak et al.	Küntscher/Street-Hansen/AO	25	8–17	N/A	120	N	2b
Gregory et al.	Küntscher/R-T	10	11.4–16.1	N/A	8	N	2b
Gonzalez-Herranz et al.	Küntscher	22	3–14	N/A	75.6	Y	2b
Beaty et al.	R-T	30	10–15	N/A	23	Y	2b
Galpin et al.	G-K/AO	22	11–16	N/A	32.9	Y	2b
Maruenda-Paulino et al.	Küntscher	29	7–16	N/A	78	Y	2b
Timmerman et al.	Küntscher/AO/G-K	22	10–14	N/A	26.7	Y	2b
Reeves et al.	Küntscher/AO	33	11–16.8	N/A	N/A	N	2b
Herndon et al.	Küntscher/interlocking	16	11–16	N/A	16	N	2b
Ziv et al.	Küntscher	8	6–12	N/A	94.8	N	2b
Valdserri et al.	Küntscher	17	6–12	N/A	78	N	4
Kirby et al.	Küntscher	13	10.8–15.6	N/A	13	N	2b

*S&N* Smith and Nephew, *R-T* Russell–Taylor, *G-K* Grosse–Kempf

The Küntscher nail has been used for the fixation of femoral fractures in children [19, 24, 25, 37], but resulted in growth disturbance [19, 24]. This prompted some authors to question the use of the Küntscher nail in children [19]. There are numerous biomechanical studies [38–40] of this implant in the literature, but none of them focus on femoral fractures in children. Furthermore, biomechanical testing indicated that the Küntscher nail did not provide adequate stability for comminuted fractures in torsion and compression [39]. Hence, the use of Küntscher nails was largely abandoned in favour of new nail designs.

With the introduction of interlocking, second-generation nails such as the Grosse–Kempf nail [14, 32, 41], the Russell–Taylor nail, and its delta version (with a triangular section, a thicker wall and a thinner diameter) were used in adolescents [28, 42, 43]. However, the indication for their use in adolescents was simply an extension of the indication for their use in adults, even though there was only a limited understanding of the intricate vascularity of proximal femoral epiphysis in adolescents. This resulted in the growth disturbances noted in patients in these series [42, 43]. The current literature has descriptions of different rigid intramedullary nails, such as the Street–Hansen nail [44], tibial nail [45, 46], and flexible interlocking intramedullary nail (FIIN) [47]. However, these represent a limited series, as the use of such devices at other centres has not been reported.

Avascular necrosis of the femoral head, limb length discrepancy (>2 cm), and malunion >10° are significant complications that have been associated with intramedullary nail fixation of adolescent femoral fractures [9, 11, 15, 17, 19]. In order to analyse the heterogeneous data in the current literature, we collated the reported complications for the different intramedullary nails (Table 3). The early-generation nails (Küntscher/Grosse–Kempf) were noted to have high rates of limb length discrepancy (5–9 %). It is interesting that AVN was not reported in the earlier series [24, 37, 48]. However, this may be due to a combination of a limited understanding of this condition and a lack of widespread availability of investigative tools such as magnetic resonance imaging (MRI) at the time. The reporting of subclinical AVN in the later series [16, 42] using MRI is indicative of this development. Results obtained with Russell–Taylor nails show good improvement, with a lower incidence of limb length discrepancy (2.8 %). It has been reported that, by and large, multiplanar nails are not associated with such complications. Two studies [34, 36] have reported on AVN and limb length discrepancy, but a lack of detail regarding the type or design of the nail used limits their interpretation.

**Table 3:** Incidence rates of major complications, obtained from the collated results of different studies

Implant	Total number of nails from different studies	Avascular necrosis (*n*, %)	Limb length discrepancy >2.0 cm (*n*, %)	Malunion >10° (*n*, %)
First generation				
Küntscher nail	138	N/R	(8, 5.8 %)	N/R
Second generation				
Grosse–Kempf nail	22	N/R	(2, 9 %)	N/R
Russell–Taylor femoral nail	118	(1, 0.85 %)	(2, 1.70 %)	N/R
Third generation				
AO femoral nail	77	(1, 1.3 %)	(2, 2.6 %)	N/R
Solid Ti nail	57	(2, 2.6 %)	N/R	N/R
Zimmer titanium nail	15	N/R	N/R	N/R
Current multiplanar nails				
ALFN	22	N/R	N/R	N/R
Sirus femoral nail	22	N/R	N/R	N/R
FIIN; Biomet	58	N/R	N/R	N/R
Miscellaneous				
Humeral nail; Smith and Nephew	95	N/R	N/R	N/R
Humeral nail; AO	24	N/R	N/R	N/R
Street–Hansen	4	N/R	N/R	N/R
Russell–Taylor tibial nail	9	N/R	N/R	N/R
Implant not stated (Ramseier et al.)	37	N/R	(1, 2.7 %)	(1, 2.7 %)
Implant not stated (Stans et al.)	13	(1, 7.7 %)	N/R	N/R

*N/R* not reported

## Discussion

The recent consensus on the age limit for rigid intramedullary fixation is ≥11 years [9, 11], although some authors advocate their use in children ≥9 years [49]. This is largely due to the increased complication rate reported following the use of flexible nails in this subset of patients [50]. Adolescents ≥49 kg have poor outcomes following other modalities of treatment for femoral fractures [11, 51], so these heavier adolescents are better managed with a rigid, locked intramedullary nail [9, 49, 52]. Excessive weight has also been shown to be an independent predictor of increased postoperative complications [51].

Attempts have been made to modify existing adult interlocking nails to overcome the limitations of the initial rigid uniplanar nail design [53]. Multiplanar nails, unlike uniplanar nails, allow a greater degree of freedom in choosing the entry point for nail insertion [21, 22, 52]. Earlier studies with piriformis entry had patients with complications such as avascular necrosis [16], coxa valga, and growth arrest of the greater trochanter [19]. Subsequent studies of nail designs with a lateral entry point report no such complication [21, 52]. Hence, the entry point of the intramedullary nail has been a subject of much debate due to the potential impact on the vascularity of the femoral head [9] and malalignment or iatrogenic fractures [54]. The entry point is largely dictated by the type/design of the nail [53]. Recent nails [21, 22, 27] have a multiplanar/helical design to avoid the piriformis entry point, which has been shown in a recent systematic review to be associated with a higher rate of avascular necrosis [55]. Specific paediatric nails are a welcome development and are suggested by some authors to be safe in skeletally immature patients younger than 12 years [56]. However, it should be noted that the results from this study were preliminary.

The design of these devices has evolved from the initial Küntscher nail to the current multiplanar rigid nails which allow introduction through a lateral entry portal. The majority of the improvements in this area have resulted from a better understanding of the femoral anatomy, in terms of both bony architecture [57, 58] and the vascularity of the physes [59]. Implant material and metallurgy is another interesting aspect of research aimed at developing devices that are fatigue resistant and possess superior biomechanical properties [60]. Multiplanar or helical design intramedullary nails are examples of this new development [22, 27, 61]. However, biomechanical studies and (more crucially) long-term clinical results of using such nails are lacking in the current literature [11].

The salient points from the current literature review point to the optimal features of an ideal rigid intramedullary nail for use in an adolescent and the subsequent management of such patients in order to achieve a good outcome. These are summarised below:

- In terms of the design, multiplanar nail curvature is desirable in order to get a close match to the femoral anatomy, along with well-designed locking screws/bolts [22, 27, 46].
- The optimal material is a titanium-based alloy with fatigue-resistant properties [27, 46].
- The entry point should be the lateral aspect of the trochanter to minimise iatrogenic damage to the femoral head vasculature and avascular necrosis [55].
- Postoperative review should pay careful attention to the recognition of major complications such as avascular necrosis to prevent morbidity [11]. When there is a diagnostic dilemma in cases with subtle and subclinical findings, MRI can be a useful investigative tool to facilitate a diagnosis [16, 42].
- The duration of follow-up should be sufficient to identify asymptomatic cases [16, 41]. There is a lack of consensus regarding the maximum length of time [11], but a minimum duration of 24 months is useful for identifying cases of late AVN [16, 36, 41, 62].
- Implant-related problems can be multifactorial. In cases with mechanical failure or iatrogenic complications secondary to the nail, nail removal and appropriate remedial procedures should be performed [9, 16, 63]. Adequate callus formation and disappearance of the fracture lines in the radiographs are simple yet useful indicators of sufficient bone healing to allow implant removal [64]. Implant removal following adequate fracture healing has also been suggested, as it is helpful to restore the normal bone mineral density in adolescents [65]. Early removal of the implant should be avoided.

In conclusion, rigid intramedullary nail fixation of femoral fractures in adolescents is a useful procedure with good clinical results. Standardisation of terminology and outcome parameters for intramedullary nail fixation in adolescents is required. Further evidence in terms of long-term clinical data and biomechanical studies is needed to be able to improve on the design of intramedullary nails in current use.

Appropriate patient selection, meticulous surgical technique, and nails that are purpose-built to avoid iatrogenic damage to the physes are key to achieving a good long-term clinical outcome.
